# Effect of interval between neoadjuvant chemoradiotherapy and surgery on disease recurrence and survival in rectal cancer: long-term results of a randomized clinical trial

**DOI:** 10.1093/bjsopen/zrac107

**Published:** 2022-10-18

**Authors:** Erhan Akgun, Cemil Caliskan, Osman Bozbiyik, Tayfun Yoldas, Basak Doganavsargil, Serdar Ozkok, Timur Kose, Bulent Karabulut, Nevra Elmas, Omer Ozutemiz

**Affiliations:** Department of General Surgery, Ege University School of Medicine, Izmir, Turkey; Department of General Surgery, Ege University School of Medicine, Izmir, Turkey; Department of General Surgery, Ege University School of Medicine, Izmir, Turkey; Department of General Surgery, Ege University School of Medicine, Izmir, Turkey; Department of Pathology, Ege University School of Medicine, Izmir, Turkey; Department of Radiation Oncology, Ege University School of Medicine, Izmir, Turkey; Department of Biostatistics, Ege University School of Medicine, Izmir, Turkey; Department of Medical Oncology, Ege University School of Medicine, Izmir, Turkey; Department of Radiology, Ege University School of Medicine, Izmir, Turkey; Department of Gastroenterology, Ege University School of Medicine, Izmir, Turkey

## Abstract

**Background:**

The optimal timing of surgery following chemoradiotherapy (CRT) is controversial. This trial aimed to assess disease recurrence and survival rates between patients with locally advanced rectal adenocarcinoma (LARC) who underwent total mesorectal excision (TME) after a waiting interval of 8 weeks or less (classic interval; CI) *versus* more than 8 weeks (long interval; LI) following preoperative CRT.

**Methods:**

This was a phase III, single-centre, randomized clinical trial. Patients with LARC situated within 12 cm of the anal verge (T3–T4 or N+ disease) were randomized to undergo TME within or after 8 weeks after CRT.

**Results:**

Between January 2006 and January 2017, 350 patients were randomized, 175 to each group. As of February 2022, the median follow-up time was 80 (6–174) months. Among the 322 included patients (CI, 159; LI, 163) the cumulative incidence of locoregional recurrence at 5 years was 10.1 per cent in the CI group and 6.9 per cent in the LI group (*P* = 0.143). The cumulative incidence of distant metastasis at 5 years was 30.8 per cent in the CI group and 18.6 per cent in the LI group (sub-HR = 1.78; 95 per cent c.i. 1.14 to 2.78, *P* = 0.010). The disease-free survival (DFS) in each group was 59.7 and 69.9 per cent respectively (*P* = 0.157), and overall survival (OS) rates at 5 years were 73.6 *versus* 77.9 per cent (*P* = 0.476).

**Conclusion:**

Incidence of distant metastasis decreased with an interval between CRT and surgery exceeding 8 weeks, but this did not impact on DFS or OS.

**Registration number:**

NCT03287843 (http://www.clinicaltrials.gov).

## Teaser

The optimal timing of surgery following preoperative chemoradiotherapy (CRT) for rectal cancer is controversial. This trial compared oncological results obtained after an interval of 8 weeks or less *versus* more than 8 weeks. This study was a phase III, single-centre, randomized, parallel-group (1:1 ratio) clinical trial. In conclusion, the incidence of distant metastasis significantly decreased with an interval between CRT and surgery exceeding 8 weeks.

## Introduction

In conjunction with the use of total mesorectal excision (TME), chemoradiotherapy has reduced local recurrence and improved long-term survival in patients with rectal cancer^1^. Randomized trials have demonstrated superior local control, lower toxicity, and better compliance with neoadjuvant radiotherapy (RT) or chemoradiotherapy (CRT) compared with adjuvant treatment^2–4^. The two main RT approaches for rectal cancer are short-course RT and long-course chemoradiotherapy (preoperative CRT); the latter is used more commonly for locally advanced rectal cancer (LARC). Randomized studies comparing these approaches have reported no significant differences in local control and survival^5–7^; however, preoperative CRT yields a higher tumour regression rate and the possibility of a pathological complete response (pCR) rate. The delayed waiting intervals were 4–8 weeks after preoperative CRT in all the randomized studies^5–8^.

Oncological outcomes are better in patients with pCR. Several studies with different treatment protocols have been conducted, comparing^9–15^ waiting intervals after preoperative CRT to increase pCR. In the Lyon R 90-01 study, the rates of clinical tumour response and pathological stage regression were higher with waiting intervals of 6–8 weeks compared with 2 weeks^16^. Although no significant difference in locoregional recurrence (LR) or overall survival (OS) on long-term follow-up was found between the groups^17,18^, the waiting interval of 6–8 weeks gained wide acceptance. Recently, studies have demonstrated higher rates of pCR, stage regression, and especially higher disease-free survival (DFS) with longer (more than 8 weeks) waiting intervals^19,20^. In the randomized study GRECCAR-6 (comparing 7 and 11 weeks), no significant difference in pCR rates was found, whereas a significant increase in morbidity and decrease in TME quality were observed in the 11-week interval group^21^. There were no significant differences in 3-year OS, DFS, and recurrence rates between the groups^22^. The randomized study by Terzi *et al.*^23^ reported extending the interval between CRT and surgery from 8 to 12 weeks resulted in a two-fold increase in pCR rate without any difference in mortality and morbidity. The oncological results of the trial are not yet known. There remains ongoing discussion regarding the optimal timing of surgery following preoperative CRT.

The aim of this study was to present the long-term oncological outcomes (disease recurrence, DFS, and OS rates) in patients with LARC who underwent TME after a waiting interval of 8 weeks or less (classic interval (CI), 28–56 days) and those who underwent curative TME after a waiting interval of more than 8 weeks (long interval (LI), 57–84 days) following preoperative CRT^24^.

## Methods

### Study design and patients

This RCT involved patients treated at the Ege University Faculty of Medicine Hospital (Izmir, Turkey) between January 2006 and January 2017.

**Fig. 1:**
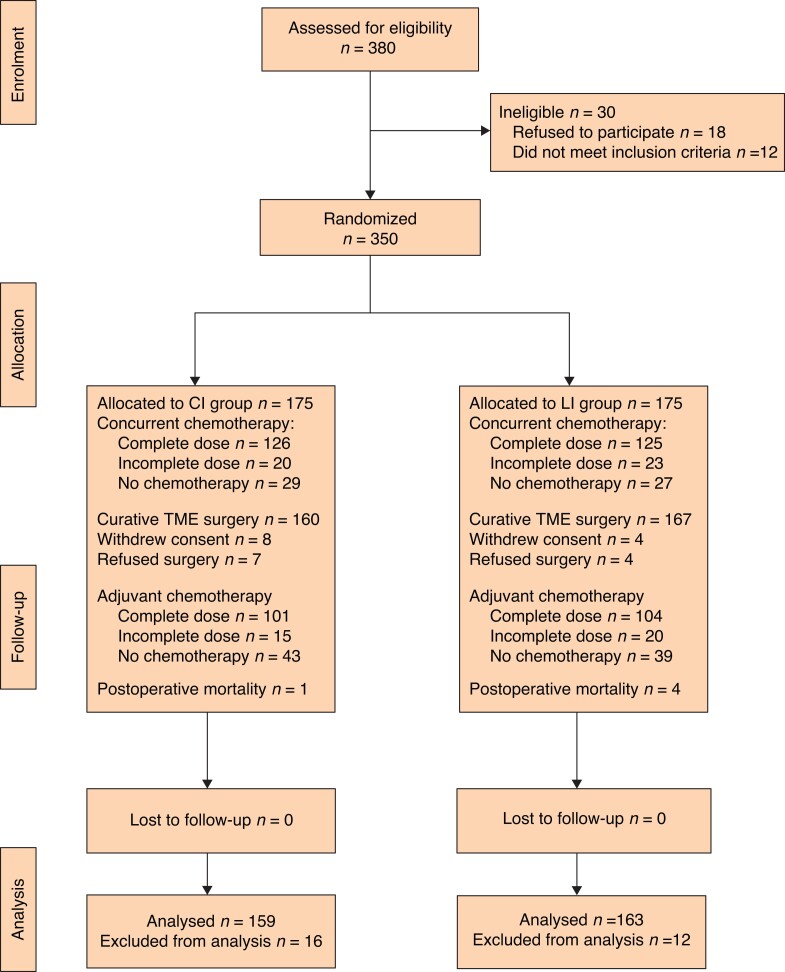
CONSORT flow diagram Classic interval group (28–56 days), long interval group (57–84 days). CI, classic interval; LI, long interval; TME, total mesorectal excision.

This study was a phase III, single-centre, randomized, parallel-group (1:1 ratio), clinical trial, and details of the study have been described previously^24^. The study consisted of patients for whom preoperative CRT was indicated after diagnosis with LARC (T3–4 or/and N (+) disease)^25^ using a multidisciplinary approach. Patients with a histologically confirmed diagnosis of stage II–III rectal adenocarcinoma situated within 12 cm of the anal verge were included in this study. The patients were scheduled to undergo TME surgery with curative intent (*Fig. 1*).

### Exclusion criteria

Patients with stage I, stage IV, or recurrent disease; patients younger than 18 years; patients with a malignancy other than adenocarcinoma; patients who underwent palliative (R2) resection or emergency surgery; patients with poor general condition (ASA grade less than 3); patients for whom CRT was contraindicated; and patients with previous/concurrent cancer were excluded.

### Randomization

Eligible patients were randomly divided into two groups to receive curative TME after a waiting interval of less than 8 weeks (CI group; range 4–8 weeks; 28–56 days) or longer than 8 weeks (LI group; range 8–12 weeks; 57–84 days) after preoperative CRT. The treatment interval was calculated from the end of neoadjuvant therapy. Randomization was performed using an adaptive biased coin technique by two surgeons on the first day of RT^26^.

This study was approved by the local ethics committee (Ege University Ethics Committee approval number 17-4.1/13). Written informed consent was obtained from each patient by the surgeon. The study was conducted in accordance with the principles of the Declaration of Helsinki and was registered at Clinicaltrials.gov (NCT03287843).

### Procedures

The details of the CRT protocol have been previously reported^24,27^. Briefly, the total dose was 50.4 Gy with a 1.8 Gy/fraction to the gross tumour and 45 Gy to the pelvic lymph nodes. As concomitant chemotherapeutic agents, 5-fluorouracil (380 mg/m^2^) and leucovorin (20 mg/m^2^) were administered every 28 days (days 1–4) for two cycles. The patients received adjuvant chemotherapy 4 weeks after surgery. Four cycles of 5-fluorouracil (425 mg/m^2^) and leucovorin (20 mg/m^2^) were administered every 28 days (days 1–5).

All patients underwent surgery by two experienced colorectal surgeons using a standardized open TME technique. The double-staple technique was used for all anastomoses, except for very low-lying rectal tumours. A routine protective stoma was used in patients with an anastomosis level of 5 cm or less from the anal verge. Bowel preparation and prophylactic antibiotics were routinely administered.

Patients were followed every 3 months for 2 years, followed by once every 6 months until 5 years after surgery, then annually thereafter. The routine evaluation consisted of physical examination, blood tests, abdominal ultrasonography and/or CT, colonoscopy (annually), and chest X-ray.

### Outcomes

The present study assessed the long-term secondary outcome oncological results (LR, distant metastasis, DFS, and OS rates) of the CI and LI groups from the previously published study^24^.

LR refers to pelvic recurrence only (isolated local recurrence) or to both pelvic recurrence and distant recurrence (occurring simultaneously or in various intervals, whichever occurred first). The recurrence of disease outside the pelvis was considered a distant metastasis. Disease recurrence (relapse) refers LR or/and distant metastasis.

DFS was defined as the time from randomization to confirmed local recurrence, distant metastasis, or death from any cause, whichever occurred first. OS was defined as the time from randomization to death from any cause.

### Pathological examination

All pathological examinations were performed by two experienced gastrointestinal pathologists blinded to the group assignment. The resected specimens were macroscopically examined and classified according to the College of American Pathologists protocols. The initial examination was performed on a fresh sample for completeness of mesorectal dissection, which was graded according to the criteria of Quirke *et al*.^28^. The histological typing and grading of differentiation were performed according to the WHO 2010 criteria. Tumour staging was performed using the TNM staging system, which classifies the depth of tumour invasion (T), the presence of regional lymph node metastasis (N), and the presence of distant metastasis (M).

Treatment response was evaluated by a five-tiered system following Mandard *et al*.^29^. The resection was defined as R1 if the circumferential resection margin was 1 mm or less or the distal margin was 1 cm or less from the tumour.

### Statistical analysis

The original trial^24^ was powered to detect a difference in the primary endpoint (pCR rates). Based on an expected pCR rate of 13 per cent in the CI group and 26 per cent in the LI group, the trial was designed to have 80 per cent power to detect a difference in the primary endpoint using a two-sided Fisher’s exact test at the 5 per cent significance level. According to this hypothesis, approximately 316 patients were required (without dropout) for this analysis. Patients were randomly assigned to the study arms in a 1:1 ratio using an adaptive biased coin technique^26^ by two surgeons (E.A. and C.C.) on the first day of RT.

In this part of the trial, groups were compared in terms of LR, distant recurrence, OS, and DFS rates (secondary endpoints). Factors that were predicted to effect secondary endpoints were sex, age, tumour localization, type of surgery, necessity of multivisceral resection, cT category, cN category, c stage, tumour differentiation, pT category, pN category, p stage, perineural invasion, lymphovascular invasion, satellite tumour, R resection, Mandard grade, TME quality, and concomitant adjuvant CT.

The CI and LI groups were compared for OS/DFS using the Kaplan–Meier method, and the differences were evaluated using the log rank test. The Cox proportional hazards model was used to calculate HRs and 95 per cent confidence intervals (95 per cent c.i.). After analysing the effect of each prognostic factor on OS and DFS, multiple Cox regression analysis (according to the enter method, no selection method was used) was applied again in the groups.

In the competing risk analysis, groups were compared in terms of LR and distant recurrence. The effect of each prognostic factor was investigated using univariable competing risk regression (Fine and Gray method^30^, using the sub-HR (SHR)) separately in the groups. After these analyses, the multiple effects of prognostic factors were investigated using the same approach. The competing risk groups for LR and distant recurrence included all deaths.

A two-sided *P* value less than or equal to 0.05 was considered statistically significant. No interim analyses of the efficacy of secondary endpoints were performed.

## Results

In total, 380 patients were evaluated for eligibility. Thirty patients did not meet the inclusion criteria for this study. The remaining 350 patients were randomly assigned to either the CI (175 patients) or the LI (175 patients) group. *Figure 1* shows the flowchart of the randomization process. All patients underwent a complete course of RT. There was no statistically significant difference between the groups regarding concurrent CT with RT (*P* = 0.960) or adjuvant CT use (*P* = 0.696).

After preoperative and perioperative evaluations of the 350 patients who underwent CRT after randomization, 16 patients in the CI group were excluded from the study (withdrew consent, 8; refused surgery, 7; and postoperative mortality, 1) and 12 patients were excluded in the LI group (withdrew consent, 4; refused surgery, 4; and postoperative mortality, 4) (*Fig. 1*). Overall, 322 patients (159 in the CI group and 163 in the LI group) were included in the analysis. The baseline characteristics are summarized in *Table 1*. The details of the histopathological results, postoperative morbidity, and mortality have been reported elsewhere^24^. In brief, the findings suggested that the rates of pCR (10.0 *versus* 18.6 per cent; *P* = 0.027) and disease stage regression were significantly increased in patients who waited for more than 8 weeks to undergo TME. A waiting interval of 8 weeks or more had no detrimental effect on surgery quality, complications, or death (*Table 2*).

**Table 1:** Baseline characteristics and type of surgery of 322 eligible patients

	Classic interval group (*n*=159)	Long interval group (*n*=163)	*P*
Age (years), mean/median/range	60.42/61.00/22–85	61.74/64.00/32–85	0.318
Sex ratio M:F	94:65	95:68	0.729
cT category			
T2	5 (3.1)	7 (4.3)	0.438
T3	103 (65.0)	101 (62.1)	
T4	51 (31.9)	55 (33.6)	
cN category			
N0	66 (41.6)	73 (45.5)	0.284
N1	61 (38.3)	55 (33.7)	
N2	32 (20.1)	35 (20.8)	
Clinical stage			
Stage II	66 (41.5)	73 (44.8)	0.652
Stage III	93 (58.5)	90 (55.2)	
Tumour localization*			
Low (0–5 cm)	101 (63.5)	93 (57.1)	0.298
Middle (5–12 cm)	58 (36.5)	70 (42.9)	
Operation type			
LAR	94 (59.0)	101 (62.0)	0.729
APR	62 (39.1)	57 (34.9)	
Others	3 (1.9)	5 (3.1)	
Multivisceral resection			
None	148 (93.1)	156 (95.7)	0.593
Applied	11 (6.9)	7 (4.3)	

Values are *n* (%) unless otherwise indicated. LAR, low anterior resection (also includes intersphincteric resection and hand-sewn coloanal anastomosis); APR, abdominoperineal resection; Others, includes total proctocolectomy (with or without pouch) and Hartmann's procedure. *Tumour distance from anal verge measured by rigid rectoscope.

**Table 2:** Postoperative histopathological results and morbidities of patients

	Classic interval	Long interval	*P*
Stage*			0.004
0 (pCR)	16 (10)	31 (18.6)	
I	26 (16.3)	38 (22.8)	
IIA/IIB/IIC	51 (31.9)/2 (1.3)/4 (2.5)	46 (27.5)/5 (3.0)/3 (1.8)	
IIIA/IIIB/IIIC	7 (4.4)/47 (29.4)/7 (4.4)	6 (3.6)/31 (18.6)/7 (4.2)	
**Differentiation**			0.011
Well	17 (10.6)	4 (2.4)	
Moderate	124 (77.5)	141 (84.4)	
Poor/mucinous	7 (4.4)/12 (7.5)	13 (7.8)/9 (5.4)	
**T category**			0.001
T0	17 (10.6)	31 (18.6)	
T1	4 (2.5)	13 (7.8)	
T2	30 (18.8)	31 (18.6)	
T3	101 (63.1)	74 (44.3)	
T4a/T4b	3 (1.9)/5 (3.1)	14 (8.4)/4 (2.4)	
N category*			0.048
N0	100 (62.5)	122 (73.1)	
N1A/1B/1C	22 (13.8)/13 (8.1)/3 (1.9)	13 (7.8)/15 (9.0)/4 (2.4)	
N2A/2B	16 (10.0)/6 (3.8)	9 (5.4)/4 (2.4)	
**PNI (+)**	16 (10)	15 (9.0)	0.753
**LVI (+)**	6 (3.8)	8 (4.8)	0.642
**Satellite tumour (+)**	10 (6.3)	7 (4.2)	0.402
**R1 resection**	12 (7.5)	15 (9.0)	0.626
**pCR(+) Mandard 1**	16 (10)	31 (18.6)	0.027
**Mandard score**			0.161
2	37 (23.1)	36 (21.6)	
3	73 (45.6)	67 (40.1)	
4	31 (19.4)	29 (17.4)	
5	3 (1.9)	4 (2.4)	
**TME quality**			0.713
Good	144 (90.0)	149 (89.2)	
Moderate	11 (6.9)	10 (6.0)	
Bad	5 (3.1)	8 (4.8)	
**Overall morbidity**	36 (22.5)	33 (19.8)	
**(Clavien–Dindo classification)**			0.307
1	4 (2.5)	7 (4.2)	
2	15 (9.4)	14 (8.4)	
3a/3b	4 (2.5)/8 (5.0)	3 (1.8)/2 (1.2)	
4a/4b	4 (2.5)/0 (0.0)	2 (1.2)/1 (0.6)	
5	1 (0.6)	4 (2.4)	

Values are *n* (%). pCR, pathological complete response; PNI, perineural invasion; LVI, lymphovascular invasion; TME, total mesorectal excision. *P is calculated by Mann–Whitney *U* test for stage and N category; otherwise, values are calculated by chi-squared test.

### Events during follow-up

As of February 2022, the median follow-up time was 80 months (mean 82.59). The ranges of the follow-up times were 7–164 months for the CI group and 6–148 months for the LI group. The mean(s.d.) waiting time was 46.1(7.3) days in the CI group and 71.1(9.3) days in the LI group. Overall, 193 surviving patients were followed up for at least 5 years (range, 61–164 months). With the exception of five patients with postoperative mortality, 129 deaths occurred during follow-up. Seventy-three deaths were related to rectal cancer (disease progression, *n* = 69; treatment-related, *n* = 4), and 56 to other causes.

LR occurred in 30 patients: 7 had local recurrence alone, and 23 had local recurrence with distant recurrences. Sixty patients had only (isolated) distant recurrences.

### Locoregional recurrence

**Fig. 2:**
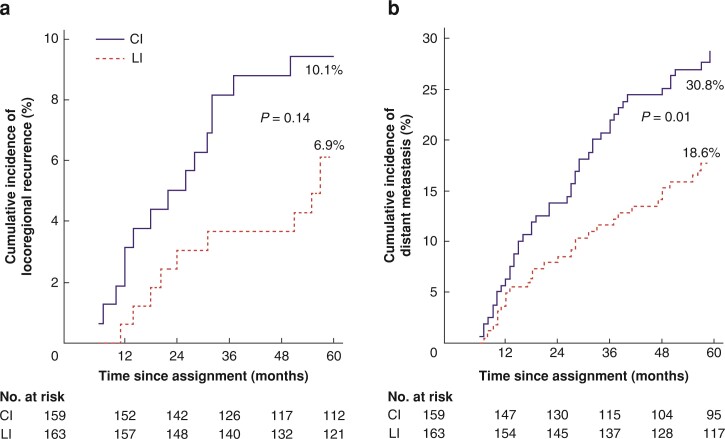
Cumulative incidences of locoregional recurrence and distant metastasis among 322 patients randomly assigned to classic interval (CI) and long interval (LI) groups using competing risk regression analysis for events **a** Locoregional recurrence. **b** Distant metastasis.

During follow-up, 19 patients from the CI group (3 patients with isolated pelvic recurrence only) and 11 patients from the LI group (4 patients with isolated pelvic recurrence only) had LR. The cumulative incidence of LR at 5 years was 10.1 per cent in the CI group and 6.9 per cent in the LI group (*P* = 0.143; *Fig. 2*). The SHR for LR in the CI group, compared with the LI group, was 1.73 (95 per cent c.i. 0.83 to 3.63).

In the CI group, univariable analyses revealed that distal tumour location (*P* = 0.017), development of postoperative complications (*P* = 0.013), and R1 resection (*P* = 0.001) were significant prognostic factors for LR. In the LI group; clinical T4 tumour (*P* = 0.025); abdominoperineal resection (*P* = 0.038); pN2 tumour (*P* = 0.013), pT4 tumour (*P* = 0.024); R1 resection (*P* = 0.033); Mandard score (*P* = 0.025); and incomplete TME (*P* = 0.015) were significant prognostic factors for the development of LR.

Multivariable analyses of the prognostic factors for LR in the CI and LI groups are presented in *Table 3*.

**Table 3:** Statistically significant prognostic factors affecting locoregional and distant recurrence (multivariable analyses)

	CI group Mult			LI group Mult		
	*P*	SHR	95% c.i.	*P*	SHR	95% c.i.
**Locoregional recurrence**						
Tumour localization						
Middle (ref)	–	–	–	–	–	–
Low	0.037	11.79	1.16–119.93	–	–	–
Mandard score						
1–2 (ref)	–	–	–	–	–	–
3	–	–	–	0.003	29.70	3.17–278.18
4–5	–	–	–	0.012	16.79	1.88–150.16
TME quality						
Good (ref)	–	–	–	–	–	–
Moderate	–	–	–	0.004	54.83	3.62–830.90
Bad	–	–	–	0.002	132.8	5.58–3162.13
**R1 resection**	0.007	3.87	1.45–10.28	–	–	–
**Postoperative complication**	0.022	3.00	1.17–7.69	–	–	–
**Distant recurrence**						
Tumour localization						
Middle (ref)	–	–	–	–	–	–
Low	0.039	1.98	1.04–3.79	–	–	–
Clinically T_4_	0.038	2.15	1.04–4.42	–	–	–
pN category						
N0 (ref)	–	–	–	–	–	–
N2	0.015	2.85	1.22–6.66	0.045	4.02	1.03–15.63
TME quality	0.044	2.97	1.03–8.61	–	–	–

CI, classic interval; LI, long interval; Mult, multivariable analysis; SHR, sub-distribution HR; TME, total mesorectal excision; ref, reference.

### Distant metastasis

During follow-up, 52 patients from the CI group (36 patients with isolated distant recurrence only) and 31 patients from LI group (24 patients with isolated distant recurrence only) had a distant recurrence. The cumulative incidence of distant metastasis at 5 years was 30.8 per cent in the CI group and 18.6 per cent in the LI group (*P* = 0.010; *Fig. 2*). The SHR for distant metastasis in the CI group compared with the LI group was 1.78 (95 per cent c.i. 1.14 to 2.78).

In the CI group, univariable analyses revealed that distal rectum tumour (*P* = 0.033); clinical T4 tumour (*P* = 0.005); pN2 tumour (*P* = 0.001); and incomplete TME were significant prognostic factors for the development of distant recurrence. In the LI group; clinical T4 (*P* = 0.025); histopathological stage III (*P*= 0.013); pN2 (*P* = 0.001); pT_4_ (*P* = 0.009); Mandard score (*P* = 0.003); R1 resection (*P* = 0.002); and incomplete TME (*P* = 0.003) were significant prognostic factors for the development of distant recurrence.

Results of multivariable analyses for both groups are presented in *Table 3*.

### Overall survival

**Fig. 3:**
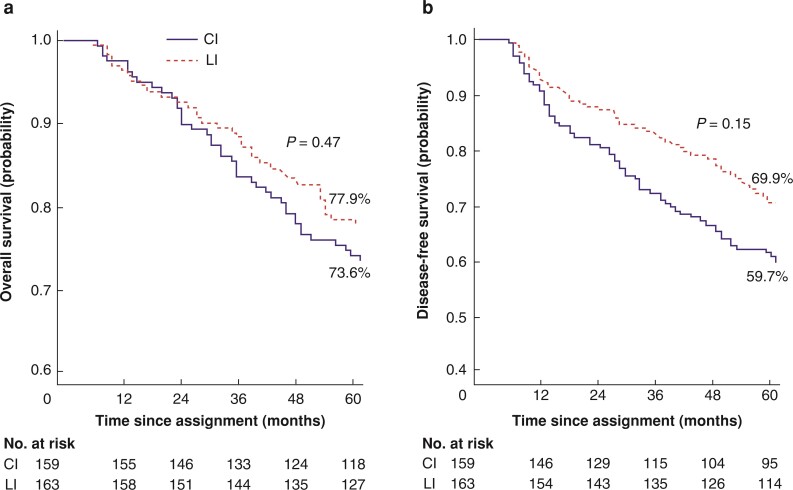
Overall survival and disease-free survival among 322 patients randomly assigned to classic interval (CI) or long interval (LI) groups using Kaplan–Meier estimation **a** Overall survival. **b** Disease-free survival.

During the follow-up interval, 75 patients in the CI group and 54 patients in the LI group died. The OS rate at 5 years was 73.6 per cent in the CI group and 77.9 per cent in the LI group (*P* = 0.476; *Fig. 3*). The HR for death in the CI group compared with the LI group was 1.14 (95 per cent c.i. 0.80 to 1.63).

In the CI group, univariable analyses revealed that age (*P* = 0.007); clinical T (*P* = 0.009); histopathological stage III (*P* = 0.014); pN2 (*P* = 0.001); R1 resection (*P* = 0.001); and incomplete TME (*P* = 0.008) were significant prognostic factors for OS. In the LI group, age (*P* = 0.005); male sex (*P* = 0.029); histopathological stage III (*P* = 0.038); pN2 (*P* = 0.038); pT4 (*P* = 0.011); R1 resection (*P* = 0.038); Mandard score (*P* = 0.012); incomplete TME (*P* = 0.035); development of postoperative complications (*P* = 0.001); and incomplete dose of adjuvant CT (*P* = 0.001) were significant prognostic factors for OS. Multivariable analyses for both groups are presented in *Table 4*.

**Table 4:** Statistically significant prognostic factors affecting disease-free and overall survival (multivariable analysis)

	CI groupMult			LI groupMult		
	*P*	HR	95% c.i.	*P*	HR	95% c.i.
**Disease-free survival**						
**TME quality**						
Good (ref)	–	–	–	–	–	–
Moderate	–	–	–	0.004	7.02	1.89–26.05
Bad	–	–	–	0.001	12.85	2.83–58.30
**Age (years)**	–	–	–	0.01	1.043	1.01–1.08
**pN category**						
N0 (ref)	–	–	–	–	–	–
N1	0.050	2.35	0.99–5.53	–	–	–
N2	0.016	3.97	1.29–12.25	–	–	–
**Male sex**	–	–	–	0.021	2.17	1.12–4.20
**Adjuvant chemotherapy**						
Complete dose (ref)	–	–	–	–	–	–
Incomplete dose	–	–	–	<0.001	6.82	2.65–17.55
None	–	–	–	0.031	2.71	1.09–6.73
**Mandard grade**						
1–2 (ref)	–	–	–	–	–	–
3	–	–	–	0.031	2.65	1.09–6.42
**Overall survival**						
**Age (years)**	0.036	1.025	1.00–1.05	0.005	1.06	1.02–1.10
**Male sex**	–	–	–	0.006	2.89	1.36–6.15
**Adjuvant chemotherapy**						
Complete dose (ref)	–	–	–	–	–	–
Incomplete dose	–	–	–	<0.001	11.19	3.64–34.45
**pN category**						
N0 (ref)	–	–	–	–	–	–
N2	0.001	3.85	1.72–8.62	–	–	–
**TME quality**						
Good (ref)	–	–	–	–	–	–
Moderate	–	–	–	0.004	10.85	2.13–55.34
Bad	–	–	–	0.001	12.32	2.70–56.23
**Mandard grade**						
1–2 (ref)	–	–	–	–	–	–
4–5	–	–	–	0.022	3.84	1.21–12.17
**Postoperative complication**						
None (ref)	–	–	–	–	–	–
Dindo–Clavien 3–4	0.019	2.62	1.17–5.87	–	–	–

CI, classic interval; LI, long interval; Mult, multivariable analysis; ref, reference; TME, total mesorectal excision.

### Disease-free survival

During the follow-up, 47 patients from the CI group died due to disease progression or treatment, 28 patients died due to other reasons, and seven patients had a relapse (LR or/and distant metastasis). In the LI group, 26 patients died due to disease, 28 patients died for other reasons, and nine patients had a relapse. The DFS rates were 59.7 per cent and 69.9 per cent in the CI and LI groups respectively (*P* = 0.157; *Fig. 3*). The HR for DFS in the CI group compared with the LI group was 1.27 (95 per cent c.i. 0.91 to 1.78).

In the CI group, univariable analyses revealed that age (*P* = 0.043); clinical T (*P* = 0.005); histopathological stage of tumour (*P* = 0.008); pN2 (*P* = 0.001); pT (*P* = 0.031); R1 resection (*P* = 0.005); and incomplete TME (*P* = 0.043) were significant prognostic factors for DFS. In the LI group, age (*P* = 0.013); male sex (*P* = 0.043); clinical T (*P* = 0.017); histopathological stage III (*P* = 0.002); pN2 (*P* = 0.003); pT (*P* = 0.004); R1 resection (*P* = 0.036); Mandard score (*P* = 0.003); incomplete TME (*P* = 0.049); and incomplete dose of adjuvant CT (*P* = 0.001) were significant prognostic factors for DFS. Multivariable analyses for both groups are presented in *Table 4*.

## Discussion

In this study, the cumulative incidence of LR at 5 years was 10.1 per cent in the CI group and 6.9 per cent in the LI group. The cumulative incidence of distant metastasis at 5 years was higher in the CI group (30.8 per cent) compared with the LI group (18.6). The DFS (59.7 *versus* 69.9 per cent) and OS rates at 5 years (73.6 *versus* 77.9 per cent) were not statistically significant.

Considering the similarities of the baseline characteristics and CRT protocols, this study suggests a significant differences in distant metastases related to the timing of surgery. Some hypotheses have been suggested to explain this.

First, extending the waiting interval significantly decreased the pT and pN category in the LI group. It has been demonstrated that the final TNM stage (p stage) is a good prognostic factor in predicting LR and distant recurrence and is a better predictor of DFS and OS than clinical stage (cTNM) before CRT^31–35^. Patients with pCR have improved oncological outcomes and this positive effect is recognized for tumours with partial response^12,36,37^. The findings that pCR and stage regression rates were higher in the LI group are not surprising, because prolonging the waiting interval is considered to lead to higher pCR rates given the biological effects of RT. DNA damage develops during irradiation, but cellular lysis occurs weeks after irradiation^21,38^. This may be accurate for the primary tumour (pT); however, lymph nodes are more complicated. In some patients with complete response in the primary tumour (pT0) after preoperative CRT, tumour cells may still be detected in the lymph nodes. Glynne-Jones *et al.*^39^ found that of 545 patients with pT0 from 47 studies, 6.6 per cent had a pN(+), with the highest rate of 17 per cent. Lymph node sterilization may occur later than in the primary tumour and it is possible to provide optimal lymph node sterilization by extending the waiting interval. In this study, detection of lymph node invasion in the 31 cases of LI group with ypT_0_ supports this (in the CI group, lymph node invasion was observed in 1 of 17 ypT_0_ cases; 5.9 per cent). The pN category was the most important prognostic factor affecting the development of distant metastasis in both groups (*Table 3*). These results are consistent with the medical literature^40–42^. Bujko *et al.*^43^ also described the association between a poor pathological response of clinically positive nodes to preoperative CRT and a high risk of distant recurrence.

Some suggest that tumour cell death occurs during treatment and not during the delay^44^. The effects of DNA damage on tumour cells are not detected morphologically until later, but the risk of recurrence will not be affected whether the surgery is delayed. It is suggested that the persistent effect of neoadjuvant treatment would continue to cause cell death over time, and consequently, waiting longer before surgery could yield less viable carcinoma at the time of surgery^45^ and it may decrease leaking of viable cancer cells thorough vascular and lymphatic channels during surgical manipulation, thereby reducing the chances of systemic recurrence^10^.

Tumour effects are generally thought to be due to cell death caused directly by DNA damage, although indirect effects such as reduction of tumour vascularity or enhanced immune recognition (immunological effects) may be important. Normal tissue effects may be acute due to direct cell death to the mucosal surface or late, due to indirect effects on the vasculature or on the stem cell component, reducing the capacity to repair future damage^46^. Extending the waiting interval may affect the clinical phase and improve oncological outcomes. There is emerging evidence that radiotherapy promotes molecular mechanisms (e.g. vascular endothelial growth factor and p53) within rectal cancer, which contribute to tumour growth, survival, and subsequent RT resistance. The extent to which this occurs may depend on the surgical interval^14,47^.

Rates of LR are significantly decreased after multimodal therapy and TME. Indicators of surgical quality include R resection status, TME quality, tumour perforation, spillage during surgery, and anastomotic leaks. These factors were compared in our previous study^24^, and no differences were detected between the groups. In the present study, even though the groups were similar regarding the rates of 5-year cumulative LR, the SHR of 1.73 may be interpreted as better local control being obtained in the LI group. The leading reason of LR was R1 resection, especially involvement of the circumferential resection margin (ICRM), which is consistent with the outcomes of the present study. Regarding LR, R1 resection was the most important prognostic factor in the CI group and was detected as a substantial factor in univariable analysis of LI group. The major prognostic factor in the LI group was TME quality. Quirke *et al.*^48^ found that both ICRM and mesorectal grading are predictors of local recurrence. Maslekar *et al.*^49^ demonstrated an association between incomplete mesorectal excision, and both local and overall recurrence. Conversely, Madbouly *et al*.^50^ claimed that in patients who received preoperative CRT and adjuvant chemotherapy, grading had no long-term prognostic value regarding recurrence unless it resulted in ICRM.

In the GRECCAR-6 study^22^ with a similar design to this study, 3-year oncological outcomes (LR, DM, OS, and DFS) were found to be similar between groups (7 *versus* 11 weeks). This may be because, contrary to the results of this study, there was no difference between the groups in terms of pTNM and pCR. The randomized clinical study of Terzi *et al.*^23^, which compared the 8 and 12-week waiting interval, demonstrated increased pCR rates in the LI group. Unfortunately, the oncological results of this study are not yet known. In conclusion, multicentred randomized clinical studies are needed to determine the optimal waiting time.

The optimal interval should facilitate maximal tumour regression, defined by maximal tumour downstaging and downsizing, with minimal risk of deterioration in the surgical outcomes, defined by low short-term morbidity (perineal and anastomotic complications) and better long-term oncological and functional outcomes^51^. In the first part of this RCT^24^, the rates of pCR and stage regression were significantly higher in the LI group than in the CI group, with similar surgical quality and postoperative morbidity. In the present part of the study, the results suggested better long-term oncological outcomes with the interval between CRT and surgery exceeding 8 weeks.

This study has limitations, including its single-centre design and the time taken to complete the study because all operations were performed by two surgeons. Another limitation is that the oncological outcomes were secondary endpoints (the study was powered to detect differences in pCR rates). The timing of surgery in the two groups (less than 8 weeks *versus* more than 8 weeks) was similar for some patients and could blunt the effect on the primary and secondary outcomes.

## Data Availability

Data are available upon reasonable request.

## References

[1] Berho M , Narang R, Van Koughnett JAM, Wexner SD. Modern multidisciplinary perioperative management of rectal cancer. JAMA Surg 2015;150:260–266 25629513 10.1001/jamasurg.2014.2887

[2] Påhlman L , Glimelius B, Graffman S. Pre- versus postoperative radiotherapy in rectal carcinoma: an interim report from a randomized multicentre trial. Br J Surg 1985;72:961–966 3910157 10.1002/bjs.1800721209

[3] Frykholm GJ , Glimelius B, Påhlman L. Preoperative or postoperative irradiation in adenocarcinoma of the rectum: final treatment results of a randomized trial and an evaluation of late secondary effects. Dis Colon Rectum 1993;36:564–572 8500374 10.1007/BF02049863

[4] Sauer R , Becker H, Hohenberger W, Rödel C, Wittekind C, Fietkau R et al Preoperative versus postoperative chemoradiotherapy for rectal cancer. N Engl J Med 2004;351:1731–1740 15496622 10.1056/NEJMoa040694

[5] Bujko K , Nowacki MP, Nasierowska-Guttmejer A, Michalski W, Bebenek M, Kryj M. Long-term results of a randomized trial comparing preoperative short-course radiotherapy with preoperative conventionally fractionated chemoradiation for rectal cancer. Br J Surg 2006;93:1215–1223 16983741 10.1002/bjs.5506

[6] Ngan SY , Burmeister B, Fisher RJ, Solomon M, Goldstein D, Joseph D et al Randomized trial of short-course radiotherapy versus long-course chemoradiation comparing rates of local recurrence in patients with T3 rectal cancer: Trans-Tasman Radiation Oncology Group trial 01.04. J Clin Oncol 2012;30:3827–3833 23008301 10.1200/JCO.2012.42.9597

[7] Erlandsson J , Holm T, Pettersson D, Berglund Å, Cedermark B, Radu C et al Optimal fractionation of preoperative radiotherapy and timing to surgery for rectal cancer (Stockholm III): a multicentre, randomised, non-blinded, phase 3, non-inferiority trial. Lancet Oncol 2017;18:336–346 28190762 10.1016/S1470-2045(17)30086-4

[8] Saglam S , Bugra D, Saglam EK, Asoglu O, Balik E, Yamaner S et al Fourth versus eighth week surgery after neoadjuvant radiochemotherapy in T3–4/N0+ rectal cancer: Istanbul R-01 study. J Gastrointest Oncol 2014;5:9–17 24490038 10.3978/j.issn.2078-6891.2013.025 PMC3904022

[9] Maas M , Nelemans PJ, Valentini V, Das P, Rödel C, Kuo LJ et al Long-term outcome in patients with a pathological complete response after chemoradiation for rectal cancer: a pooled analysis of individual patient data. Lancet Oncol 2010;11:835–844 20692872 10.1016/S1470-2045(10)70172-8

[10] Zorcolo L , Rosman AS, Restivo A, Pisano M, Nigri GR, Fancellu A et al Complete pathologic response after combined modality treatment for rectal cancer and long-term survival: a meta-analysis. Ann Surg Oncol 2012;19:2822–2832 22434243 10.1245/s10434-011-2209-y

[11] Martin ST , Heneghan HM, Winter DC. Systematic review and meta-analysis of outcomes following pathological complete response to neoadjuvant chemoradiotherapy for rectal cancer. Br J Surg 2012;99:918–928 22362002 10.1002/bjs.8702

[12] Biondo S , Navarro M, Marti-Rague J, Arriola E, Pares D, Del Rio C et al Response to neoadjuvant therapy for rectal cancer: influence on long-term results. Colorectal Dis 2005;7:472–479 16108884 10.1111/j.1463-1318.2005.00864.x

[13] Smith FM , Waldron D, Winter DC. Rectum-conserving surgery in the era of chemoradiotherapy. Br J Surg 2010;97:1752–1764 20845400 10.1002/bjs.7251

[14] Kerr SF , Norton S, Glynne-Jones R. Delaying surgery after neoadjuvant chemoradiotherapy for rectal cancer may reduce postoperative morbidity without compromising prognosis. Br J Surg 2008;95:1534–1540 18942057 10.1002/bjs.6377

[15] Kalady MF , De Campos-Lobato LF, Stocchi L, Geisler DP, Dietz D, Lavery IC et al Predictive factors of pathologic complete response after neoadjuvant chemoradiation for rectal cancer. Ann Surg 2009;250:582–588 19710605 10.1097/SLA.0b013e3181b91e63

[16] Francois Y , Nemoz CJ, Baulieux J, Vignal J, Grandjean JP, Partensky C et al Influence of the interval between preoperative radiation therapy and surgery on downstaging and on the rate of sphincter-sparing surgery for rectal cancer: the Lyon R90-01 randomized trial. J Clin Oncol 1999;17:2396–2402 10561302 10.1200/JCO.1999.17.8.2396

[17] Glehen O , Chapet O, Adham M, Nemoz JC, Gerard JP. Long-term results of the Lyons R90-01 randomized trial of preoperative radiotherapy with delayed surgery and its effect on sphincter-saving surgery in rectal cancer. Br J Surg 2003;90:996–998 12905554 10.1002/bjs.4162

[18] Cotte E , Passot G, Decullier E, Maurice C, Glehen O, François Y et al Pathologic response, when increased by longer interval, is a marker but not the cause of good prognosis in rectal cancer: 17-year follow-up of the Lyon R90-01 randomized trial. Int J Radiat Oncol Biol Phys 2016;94:544–553 26723110 10.1016/j.ijrobp.2015.10.061

[19] Tulchinsky H , Shmueli E, Figer A, Klausner JM, Rabau M. An interval >7 weeks between neoadjuvant therapy and surgery improves pathologic complete response and disease-free survival in patients with locally advanced rectal cancer. Ann Surg Oncol 2008;15:2661–2667 18389322 10.1245/s10434-008-9892-3

[20] Wolthuis AM , Penninckx F, Haustermans K, De Hertogh G, Fieuws S, Van Cutsem E et al Impact of interval between neoadjuvant chemoradiotherapy and TME for locally advanced rectal cancer on pathologic response and oncologic outcome. Ann Surg Oncol 2012;19:2833–2841 22451236 10.1245/s10434-012-2327-1

[21] Lefevre JH , Mineur L, Kotti S, Rullier E, Rouanet P, De Chaisemartin C et al Effect of interval (7 or 11 weeks) between neoadjuvant radiochemotherapy and surgery on complete pathologic response in rectal cancer: a multicenter, randomized, controlled trial (GRECCAR-6). J Clin Oncol 2016;34:3773–3780 27432930 10.1200/JCO.2016.67.6049

[22] Lefevre JH , Mineur L, Cachanado M, Denost Q, Rouanet P, De Chaisemartin C et al Does a longer waiting period after neoadjuvant radio-chemotherapy improve the oncological prognosis of rectal cancer?: three years’ follow-up results of the Greccar-6 randomized multicenter trial. Ann Surg 2019;270:747–754 31634178 10.1097/SLA.0000000000003530

[23] Terzi C , Bingul M, Arslan NC, Ozturk E, Canda AE, Isik O et al Randomized controlled trial of 8 weeks’ vs 12 weeks’ interval between neoadjuvant chemoradiotherapy and surgery for locally advanced rectal cancer. Colorectal Dis 2020;22:279–288 31566843 10.1111/codi.14867

[24] Akgun E , Caliskan C, Bozbiyik O, Yoldas T, Sezak M, Ozkok S et al Randomized clinical trial of short or long interval between neoadjuvant chemoradiotherapy and surgery for rectal cancer. Br J Surg 2018;105:1417–1425 30155949 10.1002/bjs.10984

[25] Edge S , Byrd D, Compton C, Fritz AG, Greene FL, Trotti A. AJCC Cancer Staging Manual(7th edn). New York: Springer, 2010

[26] Wei LJ . The adaptive biased coin design for sequential experiments. Ann Stat 1978;6:92–100

[27] Akgun E , Ozkok S, Tekin M, Yoldas T, Caliskan C, Kose T et al The effects of chemoradiotherapy on recurrence and survival in locally advanced rectal cancers with curative total mesorectal excision: a prospective, nonrandomized study. World J Surg Oncol 2017;15:205 29166925 10.1186/s12957-017-1275-4 PMC5700528

[28] Nagtegaal ID , Van de Velde CJH, Van Der Worp E, Kapiteijn E, Quirke P, Van Krieken JHJM. Macroscopic evaluation of rectal cancer resection specimen: clinical significance of the pathologist in quality control. J Clin Oncol 2002;20:1729–1734 11919228 10.1200/JCO.2002.07.010

[29] Mandard AM , Dalibard F, Mandard JC, Marnay J, Henry-Amar M, Pefiot JF et al Pathologic assessment of tumor regression after preoperative chemoradiotherapy of esophageal carcinoma: clinicopathologic correlations. Cancer 1994;73:2680–2686 8194005 10.1002/1097-0142(19940601)73:11<2680::aid-cncr2820731105>3.0.co;2-c

[30] Fine JP , Gray RJ. A proportional hazards model for the subdistribution of a competing risk. J Am Stat Assoc 1999;94:496–509

[31] Kim CH , Lee SY, Kim HR, Kim YJ. Pathologic stage following preoperative chemoradiotherapy underestimates the risk of developing distant metastasis in rectal cancer: a comparison to staging without preoperative chemoradiotherapy. J Surg Oncol 2016;113:692–699 26914147 10.1002/jso.24207

[32] Fokas E , Liersch T, Fietkau R, Hohenberger W, Beissbarth T, Hess C et al Tumor regression grading after preoperative chemoradiotherapy for locally advanced rectal carcinoma revisited: updated results of the CAO/ARO/AIO-94 trial. J Clin Oncol 2014;32:1554–1562 24752056 10.1200/JCO.2013.54.3769

[33] Kuo LJ , Liu MC, Jian JJM, Horng CF, Cheng TI, Chen CM et al Is final TNM staging a predictor for survival in locally advanced rectal cancer after preoperative chemoradiation therapy? Ann Surg Oncol 2007;14:2766–2772 17551794 10.1245/s10434-007-9471-z

[34] Vychnevskaia K , Dumont F, Agostini J, Julié C, Dartigues P, Lazure T et al Prognostic value of sterilized lymph nodes after preoperative chemoradiotherapy for patients with ypN0 rectal cancer. Ann Surg Oncol 2017;24:1304–1311 28008572 10.1245/s10434-016-5736-8

[35] Habr-Gama A , Perez RO, Nadalin W, Nahas SC, Ribeiro U Jr, Silva E Sousa AH Jr et al Long-term results of preoperative chemoradiation for distal rectal cancer correlation between final stage and survival. J Gastrointest Surg 2005;9:90–101 15623449 10.1016/j.gassur.2004.10.010

[36] Geva R , Davidovics H, Soyfer S, Pelles-Avraham S, Klausner JM, Inbar M et al Does residual microscopic disease after chemoradiotherapy for locally advanced rectal cancer translate into a good clinical outcome? Colorectal Dis 2017;19:237–242 27474791 10.1111/codi.13474

[37] Lee YC , Hsieh CC, Chuang JP. Prognostic significance of partial tumor regression after preoperative chemoradiotherapy for rectal cancer: a meta-analysis. Dis Colon Rectum 2013;56:1093–1101 23929020 10.1097/DCR.0b013e318298e36b

[38] Suid HD , Gallager HS. Intact tumor cells in irradiated tissue. Arch Pathol 1964;78:648–651 14208436

[39] Glynne-Jones R , Wallace M, Livingstone JIL, Meyrick-Thomas J. Complete clinical response after preoperative chemoradiation in rectal cancer: is a ‘wait and see’ policy justified? Dis Colon Rectum 2008;51:10–20 18043968 10.1007/s10350-007-9080-8

[40] Duchalais E , Glyn Mullaney T, Spears GM, Kelley SR, Mathis K, Harmsen WS et al Prognostic value of pathological node status after neoadjuvant radiotherapy for rectal cancer. Br J Surg 2018;105:1501–1509 29663352 10.1002/bjs.10867

[41] Hall MD , Schultheiss TE, Smith DD, Fakih MG, Kim J, Wong JYC et al Impact of total lymph node count on staging and survival after neoadjuvant chemoradiation therapy for rectal cancer. Ann Surg Oncol 2015;22:580–587 10.1245/s10434-015-4585-1 25956577

[42] Huebner M , Wolff BG, Smyrk TC, Aakre J, Larson DW. Partial pathologic response and nodal status as most significant prognostic factors for advanced rectal cancer treated with preoperative chemoradiotherapy. World J Surg 2012;36:675–683 22270980 10.1007/s00268-011-1409-8

[43] Bujko K , Michalski W, Kepka L, Nowacki MP, Nasierowska-Guttmejer A, Tokar P et al Association between pathologic response in metastatic lymph nodes after preoperative chemoradiotherapy and risk of distant metastases in rectal cancer: an analysis of outcomes in a randomized trial. Int J Radiat Oncol Biol Phys 2007;67:369–377 17118570 10.1016/j.ijrobp.2006.08.065

[44] Glimelius B . On a prolonged interval between rectal cancer (chemo)radiotherapy and surgery. Ups J Med Sci 2017;122:1–10 28256956 10.1080/03009734.2016.1274806 PMC5361426

[45] de Campos-Lobato LF , Geisler DP, da Luz Moreira A, Stocchi L, Dietz D, Kalady MF. Neoadjuvant therapy for rectal cancer: the impact of longer interval between chemoradiation and surgery. J Gastrointest Surg 2011;15:444–450 21140237 10.1007/s11605-010-1197-8

[46] Gwynne S , Staffurth J. Principles of cancer treatment by radiotherapy. Surg. Elsevier 2012;30:191–193

[47] Cascinu S , Graziano F, Catalano V, Staccioli MP, Rossi MC, Baldelli AM et al An analysis of p53, BAX and vascular endothelial growth factor expression in node-positive rectal cancer. Relationships with tumour recurrence and event-free survival of patients treated with adjuvant chemoradiation. Br J Cancer 2002;86:744–749 11875737 10.1038/sj.bjc.6600155 PMC2375295

[48] Quirke P , Steele R, Monson J, Grieve R, Khanna S, Couture J et al Effect of the plane of surgery achieved on local recurrence in patients with operable rectal cancer: a prospective study using data from the MRC CR07 and NCIC-CTG CO16 randomised clinical trial. Lancet 2009;373:821–828 19269520 10.1016/S0140-6736(09)60485-2 PMC2668948

[49] Maslekar S , Sharma A, MacDonald A, Gunn J, Monson JRT, Hartley JE. Mesorectal grades predict recurrences after curative resection for rectal cancer. Dis Colon Rectum 2007;50:168–175 17160574 10.1007/s10350-006-0756-2

[50] Madbouly KM , Hussein AM, Abdelzaher E. Long-term prognostic value of mesorectal grading after neoadjuvant chemoradiotherapy for rectal cancer. Am J Surg 2014;208:332–341 24581995 10.1016/j.amjsurg.2013.10.023

[51] Wasserberg N . Interval to surgery after neoadjuvant treatment for colorectal cancer. World J Gastroenterol 2014;20:4256–4262 24764663 10.3748/wjg.v20.i15.4256 PMC3989961

